# Divergent Development of Hexaploid Triticale by a Wheat – Rye –*Psathyrostachys huashanica* Trigeneric Hybrid Method

**DOI:** 10.1371/journal.pone.0155667

**Published:** 2016-05-16

**Authors:** Houyang Kang, Hao Wang, Juan Huang, Yujie Wang, Daiyan Li, Chengdou Diao, Wei Zhu, Yao Tang, Yi Wang, Xing Fan, Jian Zeng, Lili Xu, Lina Sha, Haiqin Zhang, Yonghong Zhou

**Affiliations:** 1 Triticeae Research Institute, Sichuan Agricultural University, 211 Huimin Road, Wenjiang, Chengdu, 611130, Sichuan, China; 2 Dazhou Institute of Agricultural Science, 188 Jianmin Road, Dazhou, 635000, Sichuan, China; 3 College of Resources, Sichuan Agricultural University, 211 Huimin Road, Wenjiang, Chengdu, 611130, Sichuan, China; Institute of Genetics and Developmental Biology, CHINA

## Abstract

Hexaploid triticale is an important forage crop and a promising energy plant. Some forms were previously reported for developing the hexaploid triticale, such as crossing tetraploid wheat or hexaploid wheat with rye, crossing hexaploid triticale and/or hexaploid wheat with octoploid triticale, and spontaneously appearing in the selfed progenies of octoploid triticale. In the present study, we developed an effective method for production of diverse types of hexaploid triticale via wheat—rye—*Psathyrostachys huashanica* trigeneric hybrid. Genomic in situ hybridization (GISH) and fluorescence in situ hybridization (FISH) karyotyping revealed that D genome chromosomes were completely eliminated and the whole A, B, and R genome chromosomes were retained in three lines. More interestingly, the composite genome of the line K14-489-2 consisted of complete A and B genomes and chromosomes 1D, 2R, 3R, 4R, 5R, 6R, and 7R, that of line K14-491-2 was 12 A-genome (1A-6A), 14 B-genome (1B-7B), 12 R-genome (1R-3R, 5R-7R), and chromosomes 1D and 3D, and that of the line K14-547-1 had 26A/B and 14R chromosomes, plus one pair of centric 6BL/2DS translocations. This finding implies that some of D genome chromosomes can be spontaneously and stably incorporated into the hexaploid triticale. Additionally, a variety of high-molecular-weight glutenin subunits (HMW-GS) compositions were detected in the six hexaploid triticale lines, respectively. Besides, compared with its recurrent triticale parent Zhongsi828, these lines showed high level of resistance to stripe rust (*Puccinia striiformis* f. sp. *tritici*, *Pst*) pathogens prevalent in China, including V26/Gui 22. These new hexaploid triticales not only enhanced diversification of triticale but also could be utilized as valuable germplasm for wheat improvement.

## Introduction

The small grain cereal triticale (× *Triticosecale* Wittmack), a man-made wheat—rye hybrid, is considered a promising crop due to its high genetic variation for several traits of agronomic importance. It is intended to combine the high productivity and nutritional qualities of wheat with the growth vigor and environmental tolerance possessed by rye [1–2]. Triticale is widely adapted to abiotic stress conditions such as aluminum toxicity, drought, salinity, and acidic or waterlogged soils, and also to biotic stresses including powdery mildew, leaf rust, stripe rust, stem rust, *Fusarium* head blight, scald, and leaf and glume blotch [3–7]. Originally, triticale is mainly used for forage or fodder in animal feed, serving as a good source of protein, lysine, B vitamins, and readily digested starch [8–9]. However, ongoing research indicates that triticale has some potential for use in human food consumption and remarkable improvement has been made on bread making quality during the last decades [10–11]. In recent times, environmental awareness has aroused interest in the use of triticale within bio-energy and bio-fuel production owing to its high biomass and grain yield [12–14]. Moreover, the use of triticale in the brewing industry has gained much attention [15–16].

Since the first octoploid triticale (2*n* = 8*x* = 56, AABBDDRR) has been developed by chromosome doubling of hybrids between common wheat and rye [17], thousands of primary triticale lines with a variety of ploidy levels and chromosome constitutions, including octoploid triticale (× *Triticosecale rimpaui* Wittm., AABBDDRR), hexaploid triticale (× *Triticosecale neoblaringhemii* A. Camus, AABBRR), and tetraploid triticale (× *Triticosecale semisecale* (MacKey) K. Hammer et A. Filat., DDRR) have been successfully produced [18–21]. On account of their superior meiotic stability and fertility, hexaploid triticale is deemed to be more successful than octoploid and tetraploid triticale [22–24]. Hexaploid triticale is primarily derived from direct crosses of tetraploid wheat with rye. Secondary hexaploid triticale was also synthesized by hybridizing hexaploid triticale and/or hexaploid wheat with an octoploid triticale [25]. Furthermore, many hexaploid derivatives can spontaneously emerge in the selfed progenies of octoploid triticale, with the elimination of the wheat D genome chromosomes [26–28]. Recently, Hao et al. [29] reported that some hexaploid triticale lines with complete 28 intact A/B and 14 R chromosomes and other chromosome constitutions could be rapidly produced by hybridization of synthetic hexaploid wheat with rye. Li et al. [30] developed two hexaploid triticales with great morphologic divergence derived from common wheat cultivar M8003 × Austrian rye, which contained the whole A, B, and R genome chromosomes. Kwiatek et al. [31] successfully obtained hexaploid triticale carrying leaf rust resistance gene *Lr32* via crossing triticale with the *Aegilops tauschii*–rye amphiploid.

We previously reported that the trigeneric germplasms involving *Triticum*, *Psathyrostachys* and *Secale* were successfully created by crossing wheat–*Psathyrostachys huashanica* amphiploid (PHW-SA, 2*n* = 8*x* = 56, AABBDDNsNs) with triticale (Zhongsi828, 2*n* = 6*x* = 42, AABBRR) [32]. While these observations suggest that trigeneric hybridization may be useful for triticale development, its effectiveness needs to be further evaluated [33]. The objectives of this study were to characterize the chromosome constitution of six hexaploid derivatives of wheat—rye–*P*. *huashanica* trigeneric hybrids expressing high stripe rust resistance and diverse high-molecular-weight glutenin subunits (HMW-GS) compositions by genomic in situ hybridization (GISH), fluorescence in situ hybridization (FISH), and biochemical marker.

## Materials and Methods

### Plant materials

A hexaploid triticale (×*Triticosecale* Wittmack, 2*n* = 6*x* = 42, AABBRR) line Zhongsi828, with the characteristics of large spike and grain, cold tolerance, lodging resistance, and high resistance to rust and powdery mildew, was kindly provided by Dr. LQ Zhang, Triticeae Research Institute, Sichuan Agricultural University, Sichuan, China. A wheat–*P*. *huashanica* amphiploid PHW-SA (2*n* = 8*x* = 56, AABBDDNsNs) was originally produced in our laboratory [34–35]. PHW-SA and Zhongsi828 were crossed in 2008 [32]. Then seeds selected from the F_1_ plants were bulked and advanced to the F_6_ generation by single seed descent. Six derivative lines K14-488-1, K14-489-2, K14-491-2, K14-493-1, K14-545-2, and K14-547-1, with phenotypic divergence and high resistance to stripe rust over two years of observation, were isolated from the F_6_ generation. Wheat line SY95-71 was used as a susceptible check in the tests to determine stripe rust resistance. For GISH analysis, wheat cultivar J-11 (2*n* = 6*x* = 42, AABBDD) was used as blocking DNA, and the entire genomic DNA of Chinese rye landrace “Qinling” was used as a probe.

### Meiotic pairing analysis

Meiotic pairing analysis followed the procedures described by Kang et al. [34]. Young spikes at metaphase I (MI) stage were fixed in Carnoy’s fixative (ethanol: chloroform: glacial acetic acid, 6:3:1, v/v/v) for 24 h and stored at 70% ethanol until use. The macerated root tips and anthers were squashed in 1% acetocarmine. At least 50 pollen mother cells (PMCs) were observed for each plant.

### GISH and FISH analysis

Total genomic DNA of rye landrace “Qinling” was isolated using the cetyltrimethylammonium bromide (CTAB) method [36]. Rye genomic DNA was labeled with digoxigenin-11-dUTP by a nick translation mix (Roche, Mannheim, Germany) and used as a probe in GISH. Chromosome spreads of materials, probe labeling, and in situ hybridization were carried out as previously described by Han et al. [37], with slight modifications. A total volume of 30 μL denatured hybridization solution, containing 2 × SSC, 10% dextran sulphate, 0.2% sodium dodecyl sulphate, and 1 ng/μL labeled probe DNA together with the competitor DNA, was loaded per slide and incubated for 12 h at 37°C. Finally, the chromosomes were counterstained with the propidium iodide (PI) solution (Vector Laboratories, Inc., Burlingame, USA). The in situ hybridization images were visualized using an Olympus BX-51 microscope coupled to a Photometric SenSys Olympus DP70 CCD camera.

FISH analysis was subsequently used to identify the chromosome constitution of six derivative lines, using pSc119.2 and pTa535 as probes. Probe pSc119.2 from rye repetitive sequences was used to determine the B genome chromosomes of wheat and R-genome chromosomes of rye [38]. Probe pTa535 from wheat repetitive sequences hybridizes preferentially to A and D genome chromosomes [39]. The two oligonucleotide probes were synthesized by Shanghai Invitrogen Biotechnology Co. Ltd. (Shanghai, China). Probe labeling was operated according to Tang et al. [40]. The FISH procedure was performed as described by Han et al. [37], with minor modifications. The probe mixture (4 ng/μL of each probe in 2 × SSC and 1 × TE buffer) and chromosomes were denatured together by heating for 5 min at 80°C. The chromosomes were finally counterstained with DAPI (4, 6-diamidino-2-phenylindole) solution (Vector Laboratories, Inc., Burlingame, USA). The detection and visualization of FISH patterns were the same as the aforementioned GISH protocol.

### Seed storage protein electrophoresis

HMW-GS and Low-molecular-weight glutenin subunits (LMW-GS) compositions of the six derivative lines were determined by sodium dodecyl sulfate polyacrylamide-gel electrophoresis (SDS-PAGE) following the procedure described by Yan et al. [41]. The parents PHW-SA and Zhongsi828 were used as controls in the SDS-PAGE.

### Stripe rust resistance screening

Six derivative lines, the parental species PHW-SA and Zhongsi828, and SY95-71 were evaluated for seedling and adult plant responses to stripe rust at the experimental station of Sichuan Agricultural University, Chengdu, Sichuan, China. Individual plants of each line were grown rows at 10-cm space in 30-cm wide beds and 2-m in length. The plots were surrounded by the susceptible wheat line SY95-71. Artificial inoculation was made by spraying the SY95-71 rows at the two-leaf stage with a mixture of *Pst* races CYR-32, CYR-33, V26/Gui22-9, V26/Gui22-14, Su4, and Su5 suspended in light weight mineral oil. The *Pst* races were supplied by Dr. QZ Jia, Plant Protection Institute of Gansu Academy of Agricultural Sciences, Gansu, China. Stripe rust infection type (IT) was scored based on a scale of 0, 0;, 1, 2, 3, and 4, where 0 = immunity, 0; = necrotic flecks, and 1–4 = increasing sporulation and decreasing necrosis or chlorosis. The plants scored with IT 2 or lower were considered as resistant while the plants with ITs 3 and 4 were considered as susceptible. The ITs were recorded three times when uniform severity levels were observed on susceptible check SY95-71 at booting, flowering, and milky stages [42].

## Results

### Meiotic behavior of hexaploid triticale lines

**Fig 1:**
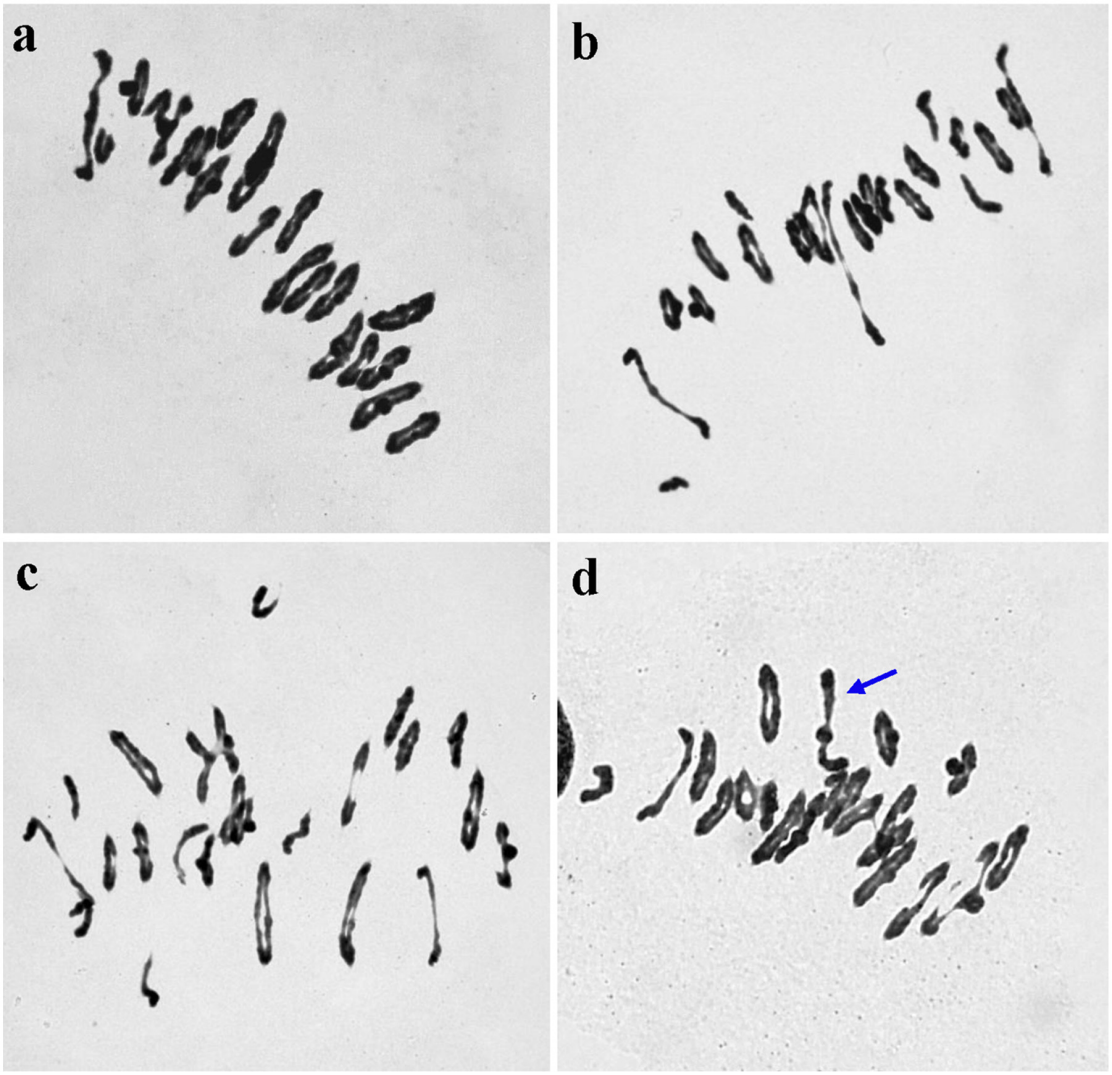
Meiotic metaphase I pairing analysis of hexaploid triticale lines. (**a)** Line K14-488-1, 2*n* = 42 = 1 II (rod) + 20 II (ring). (**b)** Line K14-489-2, 2*n* = 42 = 2 I + 4 II (rod) + 16 II (ring). (**c)** Line K14-491-2, 2*n* = 42 = 4 I + 3 II (rod) + 16 II (ring). (**d)** Line K14-547-1, 2*n* = 42 = 1 I + 1 II (rod) + 18 II (ring) + 1III (*arrow*).

Six stable derivative lines with great phenotypic divergence were obtained in 2014, namely K14-488-1, K14-489-2, K14-491-2, K14-493-1, K14-545-2, and K14-547-1. The plants of these lines grew vigorously and had a high level of seed set. These lines were selected to observe chromosome behaviors at meiotic MI, and data on chromosome pairing frequency was presented in Table 1. The results showed that all the lines with 2n = 42, the average pairing configuration was 0.94 univalents, 16.51 ring bivalents, 3.99 rod bivalents, and 0.02 trivalents per PMC. The mean number of univalents varied from 0.12 to 1.76. The number of bivalents varied from 18 to 21, and averaged 20.50 per cell (Fig 1a–1c). Trivalents were found in only one line, K14-547-1 (Fig 1d). No lagging chromosomes or chromosome bridges were observed at anaphase I and II. This indicated that the six triticale lines presented normal meiotic behavior, and they were cytologically stable.

**Table 1:** Chromosome pairing at metaphase I in the pollen mother cells of hexaploid triticale lines.

Lines	2*n*	Chromosomes pairing					
		I	II (Ring)	II (Rod)	II (Total)	III	IV
K14-488-1	42	1.36 (0–4)	16.92 (13–20)	3.40 (1–7)	20.32 (19–21)	–	–
K14-489-2	42	0.58 (0–4)	17.08 (12–21)	3.63 (0–8)	20.71 (19–21)	–	–
K14-491-2	42	1.76 (0–6)	14.20 (10–18)	5.92 (2–10)	20.12 (18–21)	–	–
K14-493-1	42	0.80 (0–4)	17.84 (15–20)	2.76 (0–6)	20.60 (19–21)	–	–
K14-545-2	42	0.12 (0–2)	16.61 (12–21)	4.33 (0–8)	20.94 (20–21)	–	–
K14-547-1	42	1.04 (0–6)	16.40 (13–18)	3.93 (1–8)	20.33 (18–21)	0.10 (0–1)	–

### GISH and FISH analysis

**Fig 2:**
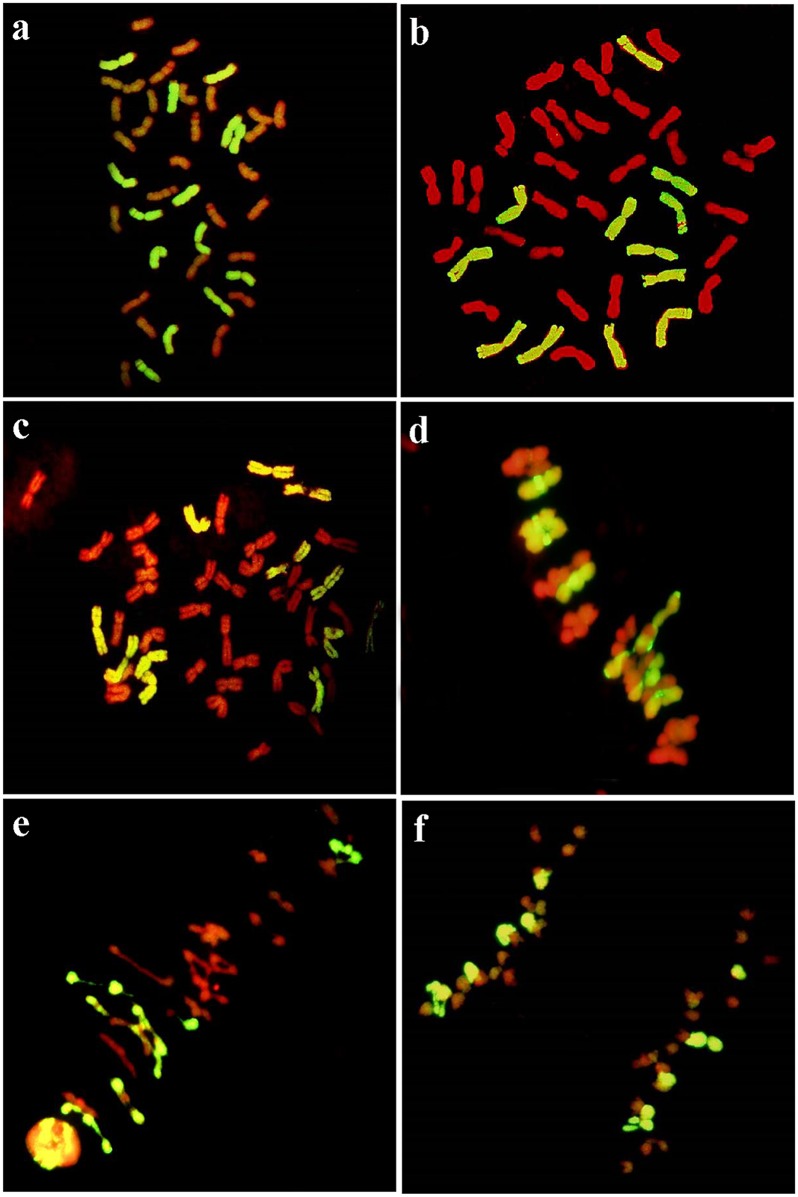
GISH identification of hexaploid triticale lines at mitotic metaphase (a–c), and meiotic metaphase I and anaphase I (d–f). The rye genomic DNA was used as probes for in situ hybridization. Chromosomes in red and yellow-green are wheat and rye chromosomes, respectively. (**a)** Line K14-488-1, 2*n* = 42 = 14R +28W. (**b)** Line K14-489-2, 2*n* = 42 = 12R +30W. **(c)** Line K14-491-2, 2*n* = 42 = 12R +30W. **(d)** Line K14-493-1, 2*n* = 42 = 14R +28W, rye chromosomes with 1 rod and 6 ring bivalents were labeled. (**e)** Line K14-489-2, 2*n* = 42 = 12R +30W, rye chromosomes with 4 rod and 2 ring bivalents were labeled. (**f)** Line K14-491-2, rye chromosomes showed regular segregation at anaphase I.

The GISH analysis was performed to determine the chromosome constitution of six derivatives using total genomic DNA of rye as the probe and J-11 total genomic DNA as the block. Lines K14-488-1, K14-493-1, K14-545-2, and K14-547-1 were found to have 14 rye chromosomes (Table 2, Fig 2a), and other lines K14-489-2 and K14-491-2 carried 12 rye chromosomes (Fig 2b and 2c). At meiosis, all these lines always contained six or seven pairs of bivalents with hybridization signals (Fig 2d and 2e), and regular segregation of each bivalent at anaphase I was also found (Fig 2f). It seems that the behavior of rye chromosomes was normal during meiosis.

**Table 2:** Chromosome constitutions of six hexaploid triticale lines.

Line	Pedigree	No. of plants tests	No. of chromosomes (chromosome constitution)
K14-488-1	PHW-SA/Zhongsi828 F_6_	5	42 (28AB + 14 R)
K14-489-2	PHW-SA/Zhongsi828 F_6_	5	42 (28AB + two 1D + 12R absent two 1R)
K14-491-2	PHW-SA/Zhongsi828 F_6_	5	42 (26AB absent two 7A + two 1D + two 3D + 12R absent two 4R)
K14-493-1	PHW-SA/Zhongsi828 F_6_	5	42 (28AB + 14 R)
K14-545-2	PHW-SA/Zhongsi828 F_6_	5	42 (28AB + 14 R)
K14-547-1	PHW-SA/Zhongsi828 F_6_	5	42 (26AB absent two 6B + two 6BL/2DS translocations+ 14 R)

A, B, D, and R mean A, B, D-genome chromosomes of wheat and R-genome chromosomes or rye, respectively

**Fig 3:**
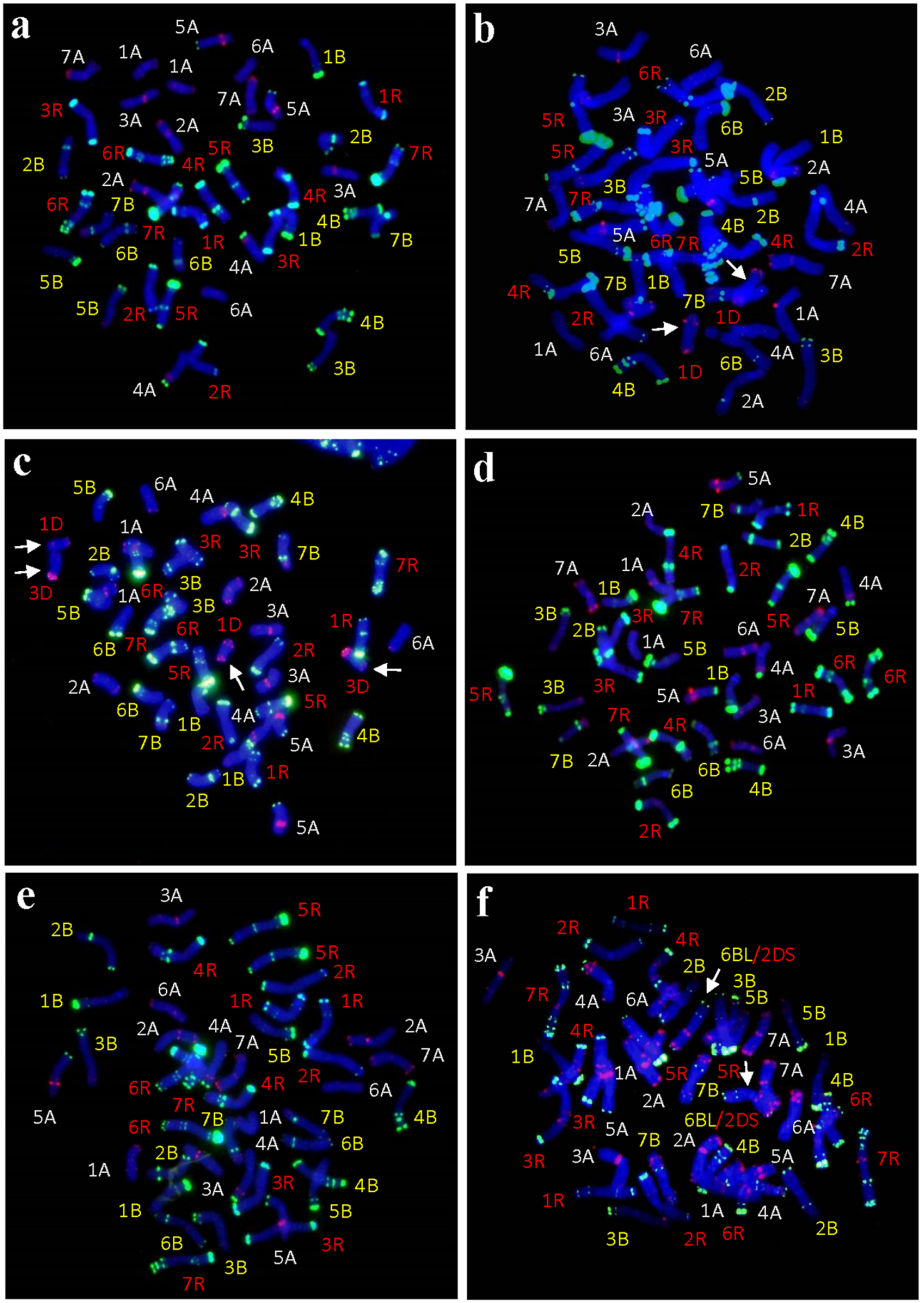
FISH karyotypes of hexaploid triticale lines using pSc119.2 and pTa535 as probes. Probes pSc119.2 and pTa535 signals were pseudo-colored as *green* and *red* in the FISH patterns, respectively. (**a)** Line K14-488-1 had complete 28 A/B and 14 R chromosomes. (**b)** Line K14-489-2 had complete 28A/B and 1D (*arrows*), 2R, 3R, 4R, 5R, 6R and 7R chromosomes. (**c)** Line K14-491-2 had 12 A (1A-6A), 14 B (1B-7B), 12 R (1R-3R, 5R-7R), and 1D and 3D (*arrows*) chromosomes. (**d)** Line K14-493-1 had complete 28 A/B and 14 R chromosomes. (**e)** Line K14-545-2 had complete 28 A/B and 14 R chromosomes. (**f)** Line K14-547-1 had 14 A (1A-7A), 12 B (1B-5B, 7B), 14 R, and a pair of 6BL/2DS translocation chromosomes (*arrows*).

Further FISH analysis showed that these derivatives could be grouped into four types with respect to their chromosome constitutions. Three lines K14-488-1, K14-493-1, and K14-545-2 displayed similar chromosome constitutions (28A/B and 14R chromosomes) as the primary hexaploid triticale between *T*. *turgidum* and rye (Fig 3a, 3d and 3e). Consequently, all the D and Ns genome chromosomes were completely eliminated during the derivation of the three hexaploid triticale lines. Line K14-489-2 consisted of six pairs of rye chromosomes and one pair of D genome chromosomes in addition to the complete A and B genomes (Fig 3b). Assuming that the FISH pattern of Chinese Spring using pSc119.2 and pTa535 as probes corresponds to the wheat cultivar used in this work, the D genome chromosomes was identified as 1D, which presented faint pTa535 signals in the terminal regions of both arms. FISH with pSc119.2 demonstrated that rye chromosomes 1R were absent in this line. More interestingly, line K14-491-2 comprised twelve rye chromosomes (Fig 2c). Reprobing with pSc119.2 and pTa535 indicated that it carried 26 A and B genome chromosomes and two pairs of D genome chromosomes (Fig 3c). One of the D genome chromosomes was identified as 1D, and another chromosome seemed to be 3D because it carried strong pTa535 signals at the terminal region of the short arm, and faint pTa535 signals in terminal region of the long arm. Probing with pSc119.2 and pTa535 revealed that rye chromosomes 4R and wheat chromosomes 7A were not included in this type (Fig 3c). Additionally, Line K14-547-1 had 14A (1A-7A), 12B (1B-5B, 7B), and 14R chromosomes, plus one pair of centric 6BL/2DS translocations (Fig 3f). Furthermore, the PMCs of lines K14-489-2, K14-491-2, and K14-547-1 showed normal meiotic behavior, possessing approximately 21 bivalents (Table 1).

### Storage protein variations detected in hexaploid triticale lines

**Fig 4:**
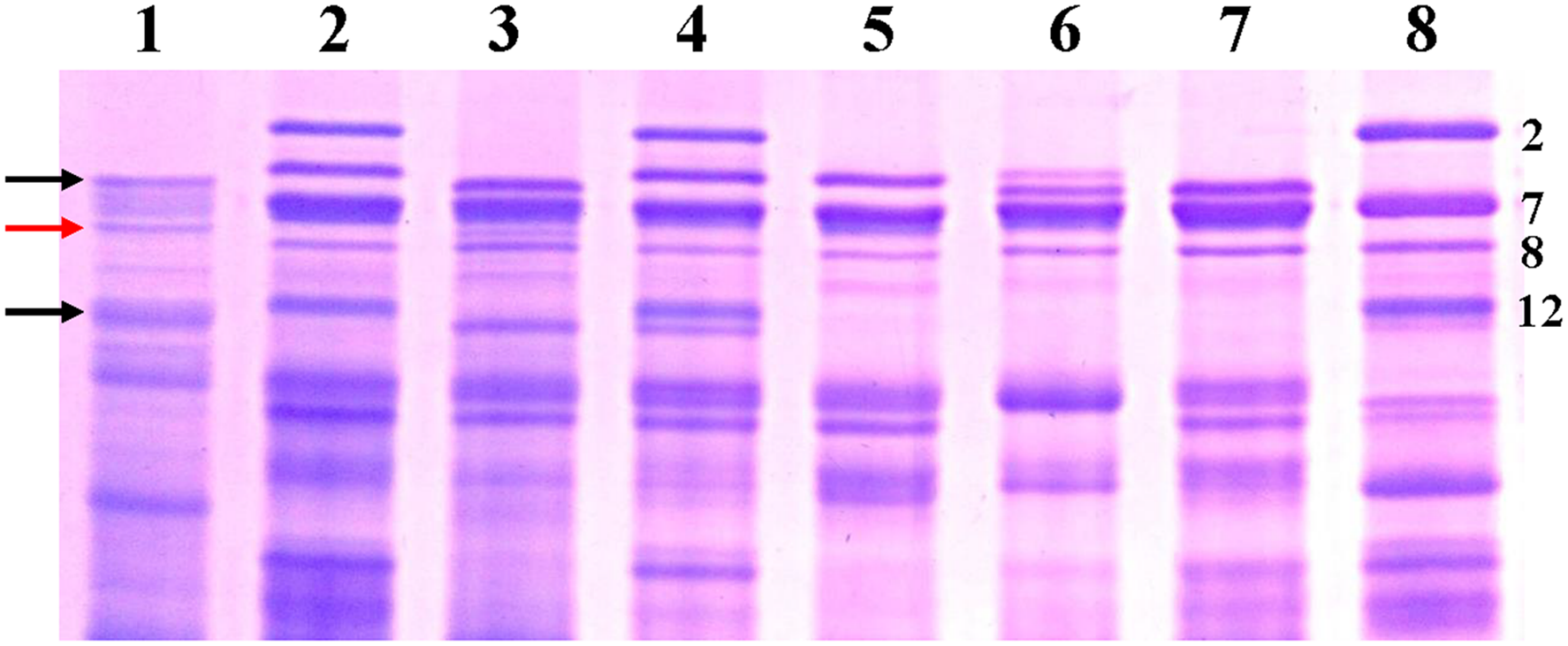
HMW-GS variations in hexaploid triticale lines and their parents. ***1*** Zhongsi828, ***2*** PHW-SA, ***3*** K14-488-1, ***4*** K14-491-2, ***5*** K14-493-1, ***6*** K14-545-2, ***7*** K14-547-1, ***8*** K14-489-2. Black arrows indicate position of inherited HMW-GS subunit of Zhongsi828. Red arrow indicates a specific band was only expressed in K14-488-1.

The HMW-GS compositions of the six hexaploid triticale lines together with their donor parents were analyzed by SDS-PAGE. As illustrated in Fig 4, six completely different HMW-GS variations were observed in these triticales. 1Dx2+1Dy12 encoded by 1D chromosome was detected in two lines K14-489-2 and K14-491-2 owing to the presence of 1D chromosome. This finding was in agreement with the FISH analyses described above. 1Bx7+1By8 encoded by 1B chromosome, which derived from the parent PHW-SA, were present at all triticale lines. In addition, not all the HMW-GS of the parent Zhongsi828 were simultaneously expressed. A single but inconsistent band was inherited in K14-488-1, K14-491-2, K14-545-2, and K14-547-1, respectively (Fig 4, indicated by black arrows), and a specific band was only expressed in K14-488-1 (Fig 4, indicated by red arrow). An exception to this was that in lines K14-493-1 and K14-489-2, all HMW-GS of the Zhongsi828 was absent. Similarly, polymorphic bands were also distinguished at these triticales in the LMW-GS region. Above results implied that multiple HMW-GS variations were discriminated in these six hexaploid triticale lines and differential expression of HMW-GS could result from mutation or expression silencing of this locus.

### Stripe rust resistance evaluation

Six hexaploid triticale lines, PHW-SA, Zhongsi828, and SY95-71were evaluated stripe rust resistance with mixture of *Pst* races CYR-32, CYR-33, V26/Gui22-9, V26/Gui22-14, Su4, and Su5 at Chengdu, Sichuan, China. At seedling and adult plant stages, Zhongsi828 and SY95-71 were susceptible to these races, showing infection types 4, respectively. PHW-SA, K14-488-1, K14-489-2, K14-491-2, K14-493-1, K14-545-2, and K14-547-1 were highly resistant to the races, all showing 0 infection type (Table 3).

**Table 3:** Infection types of hexaploid triticale lines and their parents for stripe rust with a mixture of races at seedling and adult plant stages.

Materials	No. of plants observed	Infection type	Resistance/susceptibility
PHW-SA	15	0	R
Zhongsi828	15	4	S
K14-488-1	20	0	R
K14-489-2	20	0	R
K14-491-2	20	0	R
K14-493-1	20	0	R
K14-545-2	20	0	R
K14-547-1	20	0	R
SY95-71	15	4	S

Wheat line “SY95-71” was used as susceptible control

R resistance, S susceptibility

## Discussion

### Development of hexaploid triticale by loss of most D genome chromosomes

The primary objective of this work was production of diverse chromosome constitutions (AABBDDRR, AABBRR, AABBDDNsNs, or AABBNsNs) and introgression of rye and *P*. *huashanica* chromatins into common wheat background by crossing a wheat–*P*. *huashanica* amphiploid (PHW-SA, 2*n* = 8*x* = 56, AABBDDNsNs) with hexaploid triticale (Zhongsi828, 2*n* = 6*x* = 42, AABBRR). However, after selfed generations, Xie et al. [33] found that the rye chromosome number of F_3_ lines with 2n = 41–44 ranged from 12 to 14, and only 1–2 *P*. *huashanica* chromosomes were observed in three lines (20%). In F_4_ progenies, GISH results illustrated that 21 lines of 2n = 42 (91.3%) had 12 or 14 rye chromosomes exhibiting complete pairing, and no *P*. *huashanica* chromosomes were detected. Therefore, these lines were cytologically stable during meiosis and may therefore be considered as new hexaploid triticale (unpublished data). This present study demonstrated that no octoploid triticale was found, and instead hexaploid triticale was obtained. In addition, our results revealed that these lines retained the most chromosomes of the A, B, and R genomes, while most of the D and Ns genome chromosomes were eliminated. Although we cannot exclude the possibility that some octoploid triticale was formed in F_2_ plants [32], the typically abnormal behavior of rye and wheat chromosomes of F_3_ plants must have led to their elimination during production of the subsequent generations [43]. It is known that octoploid triticale shows meiotic instability and high aneuploid frequency [22] because chromosomes from rye and wheat were eliminated [44–45]. Such chromosome loss in octoploid triticale may result in hexaploid triticale, with the retention of most of A, B, and R genome chromosomes and the elimination of most of the D genome chromosomes [26–28,46]. To date, some genetic mechanisms involved in chromosome elimination, including cytomixis-like fashion chromatin elimination from PMCs, unequal chromosome division in somatic cell, centromere loss of chromosome fragments, and chromosomal variations and asynchronous chromosome-division, were reported in octoploid triticale [29–30,45,47]. Xie et al. [43] indicated that chromosome elimination in early progenies of PHW-SA/Zhongsi828 may associate with chromatid lagging, fragmentation and micronucleation, or the immobilization of certain univalents during meiosis instead of mitosis in the relatively advanced generations. Therefore, this mechanism permitted the generation of various hexaploid triticale lines in this study.

### D genome chromosomes can be stably incorporated into the hexaploid triticale

Although the stability of the D genome is more strongly affected by the R genome in the octoploid triticale, comparing to the A and B genomes of common wheat, the more researches suggested that hexaploid triticale contained D genome chromosomes in lower frequency. Lukaszewski and Gustafson [22,48] reported very frequent 2D (2R) substitution in secondary hexaploid triticale produced by crosses between hexaploid triticale and hexaploid wheat. Tams et al. [49] and Leonova et al. [50] demonstrated that chromosomes 2D and 7D were detected in some European hexaploid triticale by SSR markers. Using FISH to characterize the chromosome constitution of 14 hexaploid lines derived from octoploid triticale, Dou et al. [27] revealed that all of the lines showed 2D (2R) substitution, and additionally two showed 1D (1R) and one showed 6D (6R). Since 1D, 2D, 6D, and 7D were found in these hexaploid triticale, Dou et al. [27] concluded that genes on chromosomes 3D, 4D, and 5D may promote the instability of the R genome, and the D genome and R genome are incompatible in the common wheat genetic background. However, the present study indicated that the composite genome of the line K14-489-2 consisted of complete A and B genomes and chromosomes 1D, 2R, 3R, 4R, 5R, 6R, and 7R, that of line K14-491-2 was 12 A genome (1A-6A), 14 B genome (1B-7B), 12 R genome (1R-3R, 5R-7R), and chromosomes 1D and 3D, and that of the line K14-547-1 had 26A/B and 14R chromosomes, and one pair of centric 6BL/2DS translocations. Moreover, the PMCs of lines K14-489-2, K14-491-2 and K14-547-1 showed stable cytology, possessing approximately 21 bivalents, with chromosome pairing configurations of 0.58 I + 3.63 II (rod) + 17.08 II (ring), 1.76 I + 5.92 II (rod) + 14.20 II (ring), and 1.04 I + 3.93 II (rod) + 16.40 II (ring) + 0.10 III, respectively. Recently, chromosomes 2D, 5D, and 7D from the wild species *Ae*. *tauschii* were involved in the hexaploid triticale using a synthetic hexaploid wheat—rye hybrid method [29]. Kwiatek et al. [31] successfully transferred the 3D genome chromatin carrying leaf rust resistance gene *Lr32* into hexaploid triticale by crossing triticale with the *Ae*. *tauschii–*rye amphiploid. It was formerly reported that 3DS promotes homoeologous chromosome pairing, and 5D carries a promoter on each arm [51–52]. Accordingly, all these findings imply that genes on chromosomes 4D may promote the instability of the R genome, and some of D genome chromosomes can be spontaneously and stably incorporated into the hexaploid triticale. The stability of D genome chromosomes in hexaploid triticale may be affected by common wheat genetic background, rye genetic composition as well as their interaction.

### The potential value of the new hexaploid triticale for wheat improvement

The present results suggest that complete and substituted hexaploid triticale can be produced using the wheat—rye–*P*. *huashanica* trigeneric hybrid method. It is believed that one of the main breeding strategies for triticale improvement was to introduce D genome chromosomes into hexaploid triticale [22,27,29,31]. Here, we developed three hexaploid triticale lines that carried 1D, 2D, and 3D chromosomes, respectively. It is known that chromosome 1D carries the *Glu-D1* locus, which plays a major role in the bread-making quality of bread wheat [53]. The dwarfing gene *Rht8* and the photoperiodic insensitivity gene *Ppd-D1* are linked on the short arm of chromosome 2D of bread wheat and play an important role in determining the geographic adaptation of modern wheat varieties [54]. The homoeologous chromosome pairing gene *Ph2* on 3DS is one of the critical genes for maintaining genetic stability in the transfer of alien genes to wheat [51,55]. Furthermore, a number of important traits are known to be controlled by loci on these chromosomes, including grain yield and seed weight, seed dormancy, glume blotch resistance, stripe rust resistance, stem rust resistance, and leaf rust resistance [56]. Besides, a variety of HMW-GS variations were detected in these hexaploid triticale lines comparing with their recurrent triticale parent Zhongsi828. Similar phenomenon occurred in recent studies of new synthesized hexaploid triticale and hexaploid trititrigias derived from *Triticum durum* and *Thinopyrum elongatum* [21,30]. Differential expression of HMW-GS could result from gene silencing or mutation of this locus occurred among the derivatives of wheat—rye–*P*. *huashanica* trigeneric hybrids. Although these triticales were derived from the same pedigree, great morphological divergences were displayed in these lines (unpublished data). Thus, these lines might be potential materials for further quality and yield improvement of hexaploid triticale.

The occurrence of new virulent stripe rust races, such as V26/Gui 22, which are effective against all previously identified *Pst* races and have been deployed in commercial cultivars to fight predominant races of the fungus in China, represents a destructive serious threat to wheat production in the Sichuan Basin and potentially in other regions of China [57]. It is, therefore, urgent to search for and transfer novel sources of resistance and to use more effective genes to counterbalance the continuous evolution of rust pathogens in wheat breeding programs. In our work, six hexaploid triticale lines were selected and identified from PHW-SA/Zhongsi828 F_6_ generation. Compared with the parent Zhongsi828, these lines were highly resistant to prevalent Chinese *Pst* races, including V26/Gui 22. Consequently, the new triticale lines provide novel and valuable bridge resources for improving stripe rust resistance in wheat.

## Abbreviations

CTAB: Cetyltrimethylammonium bromide
FISH: Fluorescence in situ hybridization
GISH: Genome in situ hybridization
HMW-GS: High-molecular-weight glutenin subunits
LMW-GS: Low-molecular-weight glutenin subunits
PMC: Pollen mother cell
*Pst*: *Puccinia striiformis* f. sp. *tritici*
SDS-PAGE: Sodium dodecyl sulfate polyacrylamide-gel electrophoresis
